# First-principles study on structural, electronic and optical properties of halide double perovskite Cs_2_AgBX_6_ (B = In, Sb; X = F, Cl, Br, I)†

**DOI:** 10.1039/d3ra02566g

**Published:** 2023-05-30

**Authors:** Chol-Jun Yu, Il-Chol Ri, Hak-Myong Ri, Jong-Hyok Jang, Yun-Sim Kim, Un-Gi Jong

**Affiliations:** a Computational Materials Design, Faculty of Materials Science, Kim Il Sung University PO Box 76 Pyongyang Democratic People's Republic of Korea cj.yu@ryongnamsan.edu.kp

## Abstract

All-inorganic halide double perovskites (HDPs) attract significant attention in the field of perovskite solar cells (PSCs) and light-emitting diodes. In this work, we present a first-principles study on structural, elastic, electronic and optical properties of all-inorganic HDPs Cs_2_AgBX_6_ (B = In, Sb; X = F, Cl, Br, I), aiming at finding the possibility of using them as photoabsorbers for PSCs. Confirming that the cubic perovskite structure can be formed safely thanks to the proper geometric factors, we find that the lattice constants are gradually increased on increasing the atomic number of the halogen atom from F to I, indicating the weakening of Ag–X and B–X interactions. Our calculations reveal that all the perovskite compounds are mechanically stable due to their elastic constants satisfying the stability criteria, whereas only the Cl-based compounds are dynamically stable in the cubic phase by observing their phonon dispersions without soft modes. The electronic band structures are calculated with the Heyd–Scuseria–Ernzerhof hybrid functional, demonstrating that the In (Sb)-based HDPs show direct (indirect) transition of electrons and the band gaps are decreased from 4.94 to 0.06 eV on going from X = F to I. Finally, we investigate the macroscopic dielectric functions, photo-absorption coefficients, reflectivity and exciton properties, predicting that the exciton binding strength becomes weaker on going from F to I.

## Abstract 2

First-principles calculations have been performed to study structural, elastic, electronic and optical properties of all-inorganic halide double perovskites Cs_2_AgBX_6_ (B = In, Sb; X = F, Cl, Br, I) for solar cell and light emission applications.

## Graphical abstract

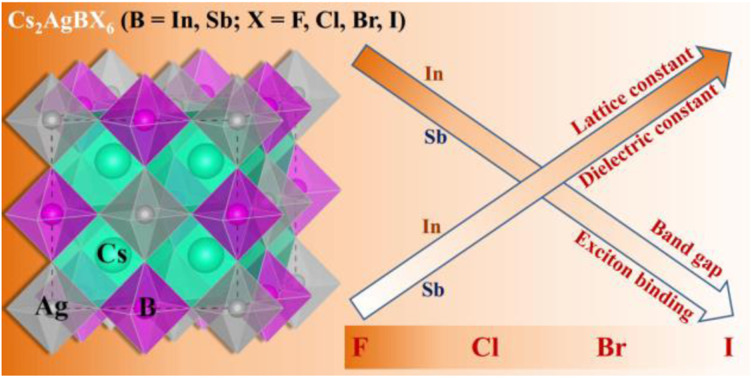

## 1 Introduction

Recently, halide perovskites have shown enormous application potential in light-emitting diodes (LEDs), photo-detectors and solar cells.^1–3^ Typically, perovskite solar cells (PSCs) adopting halide perovskites as a photo-absorber have been developed from an embryonic device^4^ into a promising next generation of solar cells^2^ with high power conversion efficiencies over 25%.^5,6^ This rapid progress is fueled by not only the excellent optoelectronic properties of halide perovskites^7,8^ but also low-cost, simple and easily-scalable fabrication of devices.^9^ However, the halide perovskites have also shown some critical problems such as instability, low efficiency and toxicity coming from the constituent elements,^10^ which are hindering their practical applications. To resolve such problems and improve the performance of perovskite-based devices, it is essential to tune the optoelectronic properties of halide perovskites. Chemical substitution is the most general method to meet this aim. In fact, the halide perovskites have a great diversity of compositions, which makes it possible to find and design advanced materials with high stability, non-toxicity and high efficiency for developing photoluminescence as well as photovoltaic applications.

Within the chemical formula ABX_3_ for single perovskites, one can conceive numerous possible combinations of cations A^+^ and B^2+^ with the halide anions X^−^ (F, Cl, Br, I) on condition that their ionic radii *r* meet the Goldschmidt tolerance factor criterion 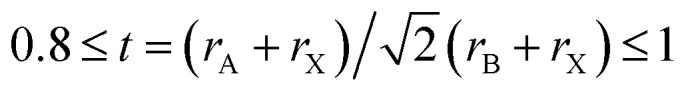 for stable phase.^11^ In particular, the B-site composition is quite flexible, thus affording halide perovskites with a broad range of metal elements at B-site including main-group (Pb, Sn, Ge, Mg, *etc.*)^12,13^ and transition-metal (Mn, Zn, Cd, Hg, *etc.*) elements.^14^ Furthermore, halide double perovskites (HDPs) with 2ABX_3_ → A_2_B_2_X_6_ stoichiometry extend accessible B-site metals, allowing for the mixed-metal HDPs A_2_BB′X_6_ (B = Au^+^, Ag^+^, Tl^+^, Na^+^, K^+^; B′ = Au^3+^, Tl^3+^, Bi^3+^, In^3+^, Sb^3+^)^15–17^ and vacancy-ordered double perovskites A_2_B□X_6_ (B = Sn^4+^, Te^4+^, Pd^4+^, *etc.*).^18,19^ The homovalent substituting Sn^2+^ or Ge^2+^ for toxic Pb^2+^ in ABX_3_ single perovskites gives rise to instability against oxidation,^20,21^ whereas the heterovalent replacement by mono- and tri-valent cations in A_2_BB′X_6_ (ref. 22) can enhance the thermal and chemical stability.^23–25^

Probably Cs_2_AgBiBr_6_, having another name of elapsolite, is the most prominent HDP with attractive optoelectronic properties and higher stability to heat and humidity compared with MAPbI_3_ (MA = methyl ammonium).^26^ Both extensive theoretical and experimental studies have shown that Cs_2_AgBiBr_6_ in cubic phase with a space group *Fm*3̄*m* has low carrier effective masses,^22^ large carrier mobilities,^27^ and long carrier recombination lifetimes.^17,28,29^ However, it has a relatively large indirect band gap of 1.8–2.2 eV^15,16,22^ and strongly localized resonant excitons with binding energy of 170 meV,^30–36^ which represent a hindrance to the photovoltaic performance as Cs_2_AgBiBr_6_-based PSCs have demonstrated a low power conversion efficiency of ∼3%.^23,25^ Filip *et al.*^16^ studied another bismuth-based double perovskite Cs_2_AgBiCl_6_ as well, finding its larger indirect band gap of 2.77 eV. Upon compression, Cs_2_AgBiBr_6_ demonstrated band gap narrowing,^37–39^ accompanied by order–disorder change of local structure which results in conversion to direct band gap nature.^40–42^ Nanostructuring or dimensional reduction has also been proved to be an effective way for tuning band gap and improving optical properties.^43–47^

Wei *et al.*^48^ synthesized antimony-based double perovskite Cs_2_AgSbBr_6_, reporting its reduced but still indirect band gap of 1.64 eV and reasonable optoelectronic properties. Such indirect feature of the band gaps limits their photovoltaic performance.^49^ Reduction and transition from indirect to direct nature of band gap can be realized by substituting thallium (Tl) for Bi as conducted by Slavney *et al.*^50^ Bismuth substitution with indium (In), resulting in other halide elapsolite Cs_2_AgInCl_6_, gives also direct wide band gap (3.3 eV) semiconducting character with favourable optoelectronic and thermoelectric properties for next generation lighting and display technologies.^1,51,52^ However, Cs_2_AgInCl_6_ suffers from low photoluminescence quantum yield (PLQY < 0.1%) because the self-trapped excitons (dominating the luminescence mechanism) and free excitons have the same orbital parity, leading to the parity-forbidden transition.^1^ To resolve such problem, Chen *et al.*^53^ suggested doping of Sc into Cs_2_AgInCl_6_ to make solid solutions of Cs_2_AgIn_1−*x*_Sc_*x*_Cl_6_, where Cs_2_AgScCl_6_ has an indirect band gap, finding that Cs_2_AgIn_0.4_Sc,0.6Cl_6_ exhibited the enhanced PL intensity (51.3%) and thermal stability.

For improving the performance of the HDP-based photo-related devices, it is required to decrease the band gap with the direct transition feature and to avoid the parity-forbidden transition. When increasing the ionic radius of halogen X as going from X = F to I, the band gaps have been found to be gradually decreased for the inorganic halide single perovskites AGeX_3_ (A = Cs, Rb)^12^ and the vacancy-ordered double perovskites K_2_SnX_6_.^19^ Therefore, it can be conceived that the solid solutions made by mixing In and Sb and/or halogen elements among X = F, Cl, Br and I in Cs_2_AgBX_6_ can meet the above requirements with further improvement of device performance. Preliminarily, it is necessary to systematically study the material properties of the In- and Sb-based HDPs Cs_2_AgBX_6_ (B = In, Sb; X = F, Cl, Br, I) with first-principles density functional theory (DFT) calculations for gaining an atomistic insight. Some of these compounds (Cs_2_AgSbCl_6_,^54^ Cs_2_AgSbBr_6_,^48^ Cs_2_AgInCl_6_ (ref. 1, 51, 52 and 54–56)) have already been synthesized experimentally and studied theoretically.^1,49,51,57^ To the best of our knowledge, however, their optoelectronic, elastic and lattice vibrational properties have not yet been investigated in the systematic way.

In this work, we aimed to obtain the comprehensive understanding of material properties of HDPs Cs_2_AgBX_6_ (B = In, Sb; X = F, Cl, Br, I) for using them as light absorbers. We first provided the structural and elastic properties, confirming that the cubic perovskite structures can be formed safely and they are mechanically stable. The phonon dispersions were also calculated to check the thermodynamic stability. Then, we considered the electronic properties that are very important for photovoltaic applications, giving the variation tendency of band gaps with halogens and the atomistic insights into transition nature with detailed analysis of electronic states. We then calculated the frequency-dependent macroscopic dielectric functions (MDFs), from which the optical properties including the photoabsorption coefficient and reflectivity were determined. Finally, we calculated the exciton properties such as exciton binding energy and exciton radius using the determined material properties in this work.

## 2 Computational method

All the DFT calculations were carried out using pseudopotential plane-wave method as implemented in the Quantum ESPRESSO (QE, version 6.5.0)^58^ and the ABINIT (version 8.8.4)^59^ packages. For the QE calculations, the ultrasoft pseudopotentials were used as provided in the Garrity–Bennet–Rabe–Vanderbilt (GBRV) library,^60^ where the valence electron configurations are Cs: 5s^2^5p^6^5d^0^6s^1^6p^0^, Ag: 4s^2^4p^6^4d^10^5s^1^5p^0^, In: 4d^10^5s^2^5p^1^, Sb: 4d^10^5s^2^5p^3^, F: 2s^2^2p^5^, Cl: 3s^2^3p^5^, Br: 4s^2^4p^5^ and I: 5s^2^5p^5^. For the ABINIT implementation, the relativistic Hartwigsen–Goedecker–Hutter (HGH) norm-conserving pseudopotentials^61^ provided in the package, where the number of valence electrons are Cs: 9, Ag: 11, In: 13, Sb: 5 and X: 7, were adopted to describe the electrostatic interaction between ionic core and valence electrons. The Perdew–Burke–Ernzerhof (PBE) functional^62^ within generalized gradient approximation (GGA) was used to treat the exchange-correlation (XC) interaction between the valence electrons. In addition, the hybrid functional in the form of Heyd–Scuseria–Ernzerhof (HSE)^63,64^ was also utilized to obtain more reasonable band gap with 80% non-local Hartree–Fock exchange addition. The spin–orbit coupling (SOC) effect was considered in the electronic band structure calculations.

**Fig. 1:**
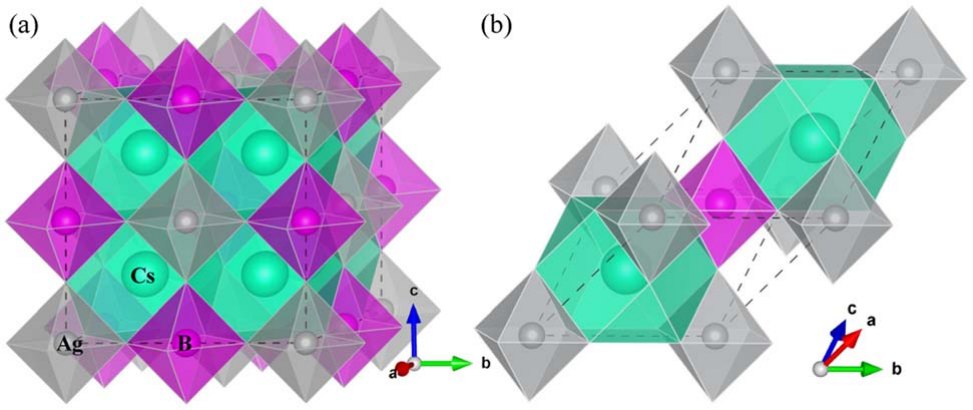
Polyhedral view of (a) conventional and (b) primitive unit cells for halide double perovskites Cs_2_AgBX_6_ (B = Sb, In; X = F, Cl, Br, I) in cubic phase with a space group of *Fm*3̄*m*. The apexes of BX_6_ octahedra and CsX_12_ dodecahedra are occupied by the halogen atoms X.

The primitive unit cell containing one formula unit (10 atoms) was used to make modeling of HDPs in cubic phase with a space group of *Fm*3̄*m*, as shown in Fig. 1. Structural optimizations were carried out using the QE package with the kinetic cutoff energies of 60 Ry and 600 Ry for wave function and electron density, respectively, and the special *k*-points of (4 × 4 × 4) mesh. All the atoms were relaxed until the atomic forces converged to 5 × 10^−4^ Ry Bohr^−1^, while the crystalline lattice was optimized until the pressure converged to 0.005 GPa. Lattice dynamics calculations were carried out using the PHONOPY^65^ package in connection with the QE code for obtaining the forces. Here, the finite-displacement approach was adopted to determine the phonon frequencies and phonon density of states (DOS) with a displacement of 0.01 Å, reduced *k*-point mesh of (2 × 2 × 2) and *q*-point mesh of (30 × 30 × 30), using the 2 × 2 × 2 supercell. The elastic constants were calculated based on the energy–strain relationship with the maximum strain of 0.05 as implemented in the ElaStic^66^ code in connection with the QE program for energy calculations.

The electronic and optical properties were determined using the ABINIT package. The optimized unit cell was used for calculations. The cut-off energy for plane wave basis set was set to 40 Ha and the special *k*-point mesh was set to (6 × 6 × 6). The electronic band structures were calculated both with the PBE-GGA and the HSE hybrid functionals for comparison. The MDFs were calculated by solving the Bethe–Salpeter equation (BSE) with the excitonic (EXC) effect and using the Haydock iterative method within the Tamm–Dancoff approximation, as implemented in the ABINIT package. In addition, the MDFs with no local field (NLF) effects were obtained with the Kohn–Sham energies and the GW energies, respectively, within the random phase approximation (RPA) for comparison.

## 3 Results and discussion

### 3.1 Crystalline lattice and elastic properties

The halide double perovskites Cs_2_AgBX_6_ (B = In, Sb; X = F, Cl, Br, I) under study were suggested to be in the face-centered cubic (*fcc*) structure with a space group of *Fm*3̄*m* (225) as confirmed in several experiments. Firstly, we checked the formability of double perovskite A_2_BB′X_6_ by evaluating the geometric factors such as the effective Goldschmidt tolerance factor *t* and the octahedral factor *μ* defined as follows,^68^1
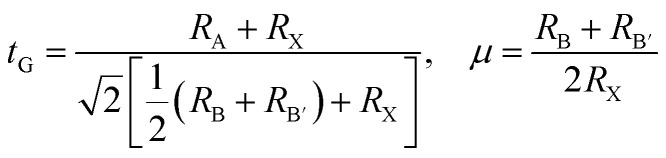
where *R*_*i*_ is the ionic radius of the *i*th-ion. The Shannon ionic radii were adopted as 1.88 Å for 12-coordinated Cs^+^, 1.15 Å for 6-coordinated Ag^+^, 0.80 and 0.76 Å for 6-coordinated In^3+^ and Sb^3+^ cations, and 1.33, 1.81, 1.96 and 2.20 Å for 6-coordinated Fe^−^, Cl^−^, Br^−^ and I^−^ anions.^69^Table 1 lists the geometric factors evaluated by using these ionic radii. It can be seen that both the *t*_G_ and *μ* values show gradual decreases from 0.985 to 0.909 and from 0.733 to 0.443 for the In-based HDPs, and from 0.993 to 0.914 and from 0.718 to 0.434 for the Sb-based HDPs, as increasing the atomic number of halogen atom going from F to I. It should be emphasized that the Goldschmidt tolerance and the octahedral factors for all the HDPs are placed in the stable ranges of 0.8 ≤ *t*_G_ ≤ 1 and 0.41 ≤ *μ* ≤ 0.9,^70^ indicating certain formations of stable perovskite structures.

**Table 1:** Geometric factors such as the Goldschmidt tolerance factor (*t*_G_) and the octahedral factor (*μ*), and the optimized lattice constants and position of halogen atoms in Cs_2_AgBX_6_ (B = In, Sb; X = F, Cl, Br, I)

Compound	Geometric factors		Lattice constant (Å)		X position *x*
	*t*_G_	*μ*	This	Prev.	
Cs_2_AgInF_6_	0.985	0.733	9.225		0.2289
Cs_2_AgInCl_6_	0.937	0.539	10.625	10.502^a^	0.2413
Cs_2_AgInBr_6_	0.925	0.497	11.175	11.20^b^	0.2443
Cs_2_AgInI_6_	0.909	0.443	12.006		0.2489
Cs_2_AgSbF_6_	0.993	0.718	9.438		0.2353
Cs_2_AgSbCl_6_	0.944	0.528	10.832	10.699^c^	0.2471
Cs_2_AgSbBr_6_	0.931	0.487	11.359		0.2491
Cs_2_AgSbI_6_	0.914	0.434	12.132		0.2512

a Experiment.^1,51,53^

b PBE calculation.^57^

c Experiment.^67^

For these HDPs, the structural optimizations were performed using the primitive unit cells to determine the crystalline lattice constants and atomic positions. In the unit cell, the Wyckoff positions of Cs, Ag, B′ and X atoms are known to be 8*c* (0.25, 0.25, 0.25), 4*a* (0, 0, 0), 4*b* (0.5, 0.5, 0.5) and 24*e* (*x*, 0, 0), respectively. In Table 1, the optimized lattice constants and position *x* of X atoms are listed in comparison with the available experimental data. For the case of Cs_2_AgInCl_6_, our calculations overestimated the lattice constant with an allowable relative error of 1.4% compared with the experiment^51^ in accordance with the general tendency of GGA-PBE calculation. As going from F to I, the lattice constant was found to gradually increase for both the In- and Sb-based HDPs due to the increase of ionic radius of halogen atom, resulting in weakening of Ag–X and B–X interactions. Accordingly, the position of halogen atom was varied systematically. The In-based HDPs have slightly smaller lattice constants than the Sb-based counterparts, although the ionic radius of In^3+^ cation (0.8 Å) is larger than that of Sb^3+^ cation (0.76 Å). This indicates that the attraction between In^3+^ cation and X^−^ anion is stronger than that between Sb^3+^ cation and X^−^ anion.

To check the formation feasibility, we calculated their elementary (*E*^e^_f_) and binary (*E*^b^_f_) formation energies as follows,

2 *E*^e^_f_ = *E*_Cs_2_AgBX_2__ − (2*E*_Cs_ + *E*_Ag_ + *E*_B_ + 3*E*_X_2__),

3 *E*^b^_f_ = *E*_Cs_2_AgBX_6__ − (2*E*_CsX_ + *E*_AgX_ + *E*_BX_3__),

where *E*_comp_ is the total energy of the corresponding compound. For each crystalline compound, all the possible phases that were available in the Inorganic Crystal Structure Database (ICSD)^71^ were considered with structural optimizations and the phase with the lowest total energy was selected (see Table S1†). For the elementary substances, Cs was in the body-centered cubic (*bcc*) phase (space group *Im*3̄*m*), Ag was in the *fcc* phase (*Fm*3̄*m*), In and Sb were in the tetragonal phase (*I*4/*mmm*), and the halogen was suggested to be gas phase (diatomic molecule). For the binary compounds, all the CsX and AgX compounds were in the *fcc* phase (*Fm*3̄*m*), except AgI which was in the zinc blende structure (*F*4̄3*m*), and all the BX_3_ (B = In, Sb) compounds were in the orthorhombic phase. Table 2 shows the calculated elementary and binary formation energies (see Table S2 and S3† for details). For all the HDPs, the formation energies were calculated to be negative, indicating that they could be formed exothermically from the binary compounds as well as the elementary substances. As going from F to I, the magnitude of *E*_f_ values was found to gradually decrease, implying that the formability can be reduced.

**Table 2:** Formation energy (*E*_f_) per formula unit (fu) in the elementary (Elem.) and binary (Bin.) forms, bulk modulus (*B*), shear modulus (*G*), Young's modulus (*E*), Poisson's ratio (*ν*), Pugh's ratio (*B*/*G*), and anisotropy factor (*A*) calculated for cubic halide double perovskites Cs_2_AgB′X_6_ (B = In, Sb; X = F, Cl, Br, I)

Compound	*E*_f_ (eV/fu)		Elastic moduli (GPa)			*ν*	*B*/*G*	*A*
	Elem.	Bin.	*B*	*G*	*E*			
Cs_2_AgInF_6_	−23.99	−1.71	73.82	35.32	91.38	0.59	2.09	1.14
Cs_2_AgInCl_6_	−14.43	−0.84	26.36	9.04	24.34	0.69	2.92	1.41
Cs_2_AgInBr_6_	−12.60	−0.49	23.23	8.11	21.79	0.69	2.86	1.26
Cs_2_AgInI_6_	−9.96	−0.03	18.80	7.61	20.11	0.64	2.47	0.67
Cs_2_AgSbF_6_	−22.64	−0.97	67.48	32.43	83.87	0.59	2.08	1.08
Cs_2_AgSbCl_6_	−13.43	−0.84	24.03	8.20	22.08	0.69	2.93	1.04
Cs_2_AgSbBr_6_	−11.88	−0.74	21.68	7.90	21.14	0.67	2.74	0.84
Cs_2_AgSbI_6_	−9.55	−0.36	18.12	6.79	18.11	0.67	2.67	0.90

**Fig. 2:**
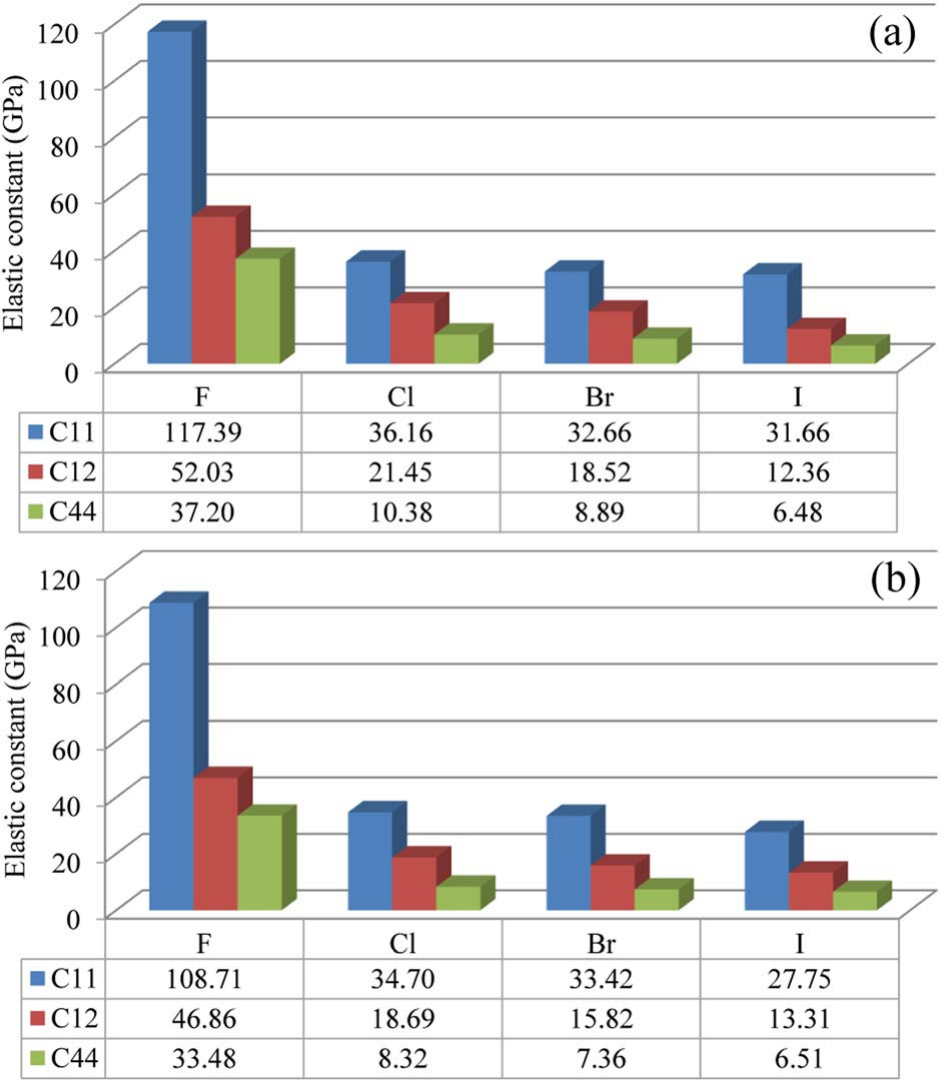
Elastic constants of *C*_11_, *C*_12_ and *C*_44_ in (a) Cs_2_AgInX_6_ and (b) Cs_2_AgSbX_6_ (X = F, Cl, Br, I) (unit: GPa).

Then, the elastic constants were calculated to assess the mechanical stability of the compounds. In the cubic structure, there are three independent components of elastic tensor *viz.*, *C*_11_, *C*_12_ and *C*_44_. Fig. 2 shows the calculated three kinds of elastic constants for the In- and Sb-based HDPs, intuitively demonstrating their systematic decreases as increasing the atomic number of halogen atom. Such decrease might be associated with the weakening of B–X and Ag–X interactions as going from F to I, as revealed by increase of lattice constant. In like wise, the In-based HDPs have larger elastic constants than the Sb-based counterparts due to the stronger B–X interaction. Nevertheless, we confirmed that the calculated elastic constants meet the mechanical stability criteria^72^ given as follows,

4 *C*_11_ > 0, *C*_44_ > 0, *C*_11_ − *C*_12_ > 0, *C*_11_ + 2*C*_12_ > 0,

which indicates that all these HDPs are mechanically stable.

Using the elastic constants, we evaluated the bulk and shear moduli within the Voigt (V), Reuss (R) and further Voigt–Reuss–Hill approximations as follows,5
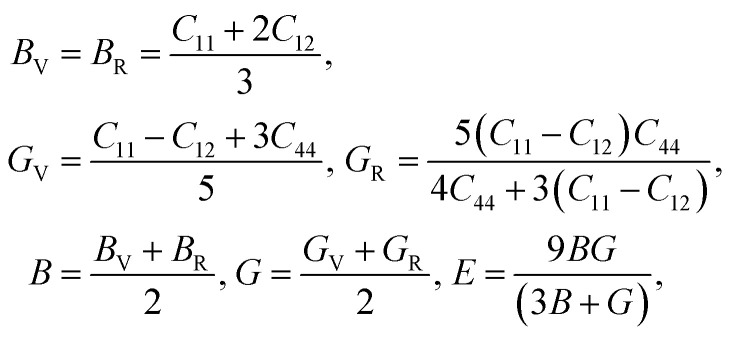
where *B*, *G* and *E* are the bulk, shear and Young's moduli. Furthermore, the Poisson's ratio *ν* and anisotropy factor *A* can be calculated as follows,6
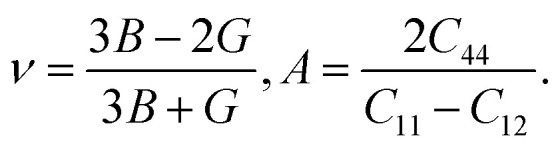


The Pugh's ratio *B*/*G* was also evaluated. Table 2 lists the calculated elastic properties for these double perovskites. The bulk, shear and Young's moduli were found to systematically decrease as going from F to I, and those of In-based perovskites were slighter larger than those of Sb-based ones. These double perovskites were identified to be surely ductile, because the calculated values of Poisson's and Pugh's ratios were far above the limiting values of 0.26 and 1.75, respectively. Meanwhile, the calculated values of anisotropy factor *A* were deviated from unity, which is for isotropic crystal, indicating that all the considered HDPs are elastically anisotropic in nature.

### 3.2 Phonon property and dynamical stability

**Fig. 3:**
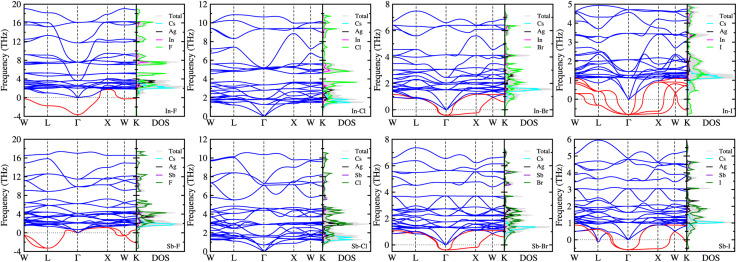
Phonon dispersion curves and phonon density of states (DOS) of halide double perovskites Cs_2_AgBX_6_ (B = In, Sb; X = F, Cl, Br, I) in cubic phase, calculated with the finite-displacement method using 2 × 2 × 2 supercells. Red lines indicate soft modes with imaginary phonon frequencies.

On the condition that all the Cs_2_AgBX_6_ perovskites were confirmed to be stable in geometric and mechanical ways, we examined the dynamical stability by calculating their phonon dispersions with the finite-displacement approach using the 2 × 2 × 2 supercells. Fig. 3 displays the calculated phonon dispersions together with the phonon DOS. For these double perovskites, the cubic structure confirms 30 phonon modes as the primitive unit cell contains 10 atoms. Among these 30 phonon modes, we identified the 3 acoustic modes, characterized by approaching zero phonon frequency as going to the zone centre *Γ*-point. The rest 27 modes were optical ones, which can be divided into low- and high-frequency modes, and imaginary-frequency soft modes especially for dynamically unstable compounds.

As can be seen in Fig. 3, the soft phonon modes (red-colored lines) were observed in the most HDPs except Cs_2_AgBCl_6_ (B = In, Sb). This indicates that only the Cl-based double perovskites are dynamically stable and other halogen-based perovskites are unstable in cubic phase. According to the recent work of Lahnsteiner and Bokdam,^73^ however, the presence of imaginary phonon modes does not necessarily imply that the structure is unstable. Based on the established fact for dynamical stability and phase transition in organic–inorganic hybrid halide perovskites^74^ or all-inorganic single^75–77^ and double halide perovskites,^78,79^ these unstable HDPs should exhibit phase transition from cubic phase, which might be only stable at high temperature, to other phases such as tetragonal, orthorhombic or monoclinic phases at lower temperature.^77,79^ In both the all-inorganic single and double perovskites, the octahedral tilting or distortion associated with the anharmonic (soft) phonon modes causes a series of phase transition as decreasing temperature. However, the phase transition series are slightly different each other; the single perovskites (*e.g.*, CsSnI_3_) show the transition of cubic (500 K) → tetragonal (380 K) → orthorhombic,^75^ whereas the double perovskites (*e.g.*, Cs_2_SnI_6_) show the transition of cubic (137 K) → tetragonal (44 K) → monoclinic.^79^

It was found that the Br-based double perovskites exhibited very weak soft phonon modes compared with the F- and I-based compounds. Through the phonon DOS analysis, it was revealed that the low-frequency phonon modes were mainly attributed to the interaction between Cs and halogen atoms, while the high-frequency phonon modes were originated from interactions between B (In or Sb) and halogen atoms. The soft phonon modes were found to be mostly contributed from halogen atoms. The contributions of Ag atoms were found in the middle-frequency regions from ∼2 to ∼4 THz for all the compounds. As going from F to I, the highest phonon frequency was found to gradually decrease from ∼18 or 17 THz to 5 or 6 THz for the In- or Sb-based HDPs. The In-based compounds exhibit slightly higher phonon frequencies for the highest mode than the Sb-based counterparts, except the I-based compounds which have severe soft modes.

### 3.3 Electronic properties

Considering that the cubic phases can be formed at high temperature for the perovskites having soft phonon modes, we investigated the electronic properties of the cubic HDPs Cs_2_AgBX_6_. We calculated the electron band structures, from which the fundamental band gap and effective masses of charge carriers of conduction electron and hole were extracted. The careful analysis of electron DOS were also provided. These properties and quantities are of great importance in understanding the light absorption and carrier transport mechanisms for solar cell applications. To overcome the limitations of semi-local GGA-PBE functional for the description of band structures (see Fig. S1, ESI†), we employed the HSE hybrid functional with 80% nonlocal Hartree–Fock exchange addition. For the inorganic halide double perovskites, the SOC effects were known to be negligible,^51^ as confirmed in this work (see Fig. S2, ESI†).

**Fig. 4:**
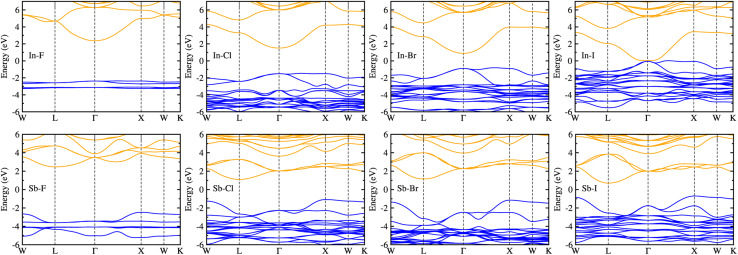
Electronic band structures along the high-symmetry points of *W*–*L*–*Γ*–*X*–*W*–*K* in the Brillouin zone for halide double perovskites Cs_2_AgBX_6_ (B = In, Sb; X = F, Cl, Br, I) in cubic phase, calculated by using the HSE hybrid XC functional with 80% nonlocal Hartree–Fock exchange addition. The blue (orange) lines indicate the valence (conduction) bands.

Fig. 4 shows the electronic band structures plotted along the high-symmetry points of *W*–*L*–*Γ*–*X*–*W*–*K* in the Brillouin zone (BZ). The In-based HDPs were found to have direct band gaps with both the valence band maximum (VBM) and conduction band minimum (CBM) located at the centre of BZ (*Γ* point). Meanwhile, the Sb-based HDPs have indirect band gaps between VBM at *X* point and CBM at *L* point. The calculated band gaps are listed in Table 3. Compared with the available experiments, the calculated direct band gaps of 3.02 eV and 1.78 eV for Cs_2_AgInCl_6_ and Cs_2_AgInBr_6_ with HSE in this work were in reasonable agreement with the experimental values of 3.3 eV (ref. 51) and 1.5 eV,^68^ respectively. Also, the calculated indirect band gaps for CsAgSbCl_6_ (3.32 eV) and CsAgSbBr_6_ (2.30 eV) were well agreed with the explicit many-body GW calculation^30^ (3.43 and 2.74 eV). These indicate that our computational settings in this work are surely reliable for electronic structure calculations for inorganic HDPs. For cases of the In-based HDPs, it should be noted that the self-trapped excitons and free excitons have the same orbital parity, leading to the parity-forbidden transition and low photoluminescence quantum yield.^1^ The effect of parity-forbidden transitions near the band edge can be estimated by calculating the emission energy through post-processing the outputs from the GW, BSE and phonon calculations. Since the main point in the present work is the variation tendency of the material properties, we avoid such computationally very demanding task and proceed the discussion with DFT calculation to make consistency in analysis.

**Table 3:** The calculated band gap in comparison with the available experimental and theoretical data, effective masses of conductive electron 
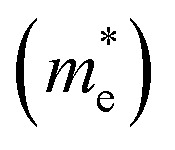
 and hole 
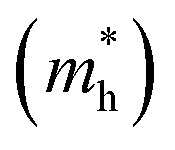
 and reduced effective mass 
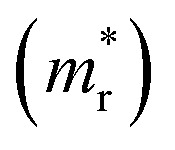
 in the unit of free electron mass (*m*_e_), static dielectric constant (*ε*_s_), and exciton properties including exciton binding energy (*E*_b_) and exciton radius 
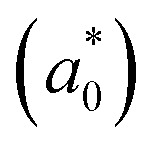
 for HDPs Cs_2_AgBX_6_ (B = In, Sb; X = F, Cl, Br, I). The values in the brackets indicate the previous data

Compound	*E*_g_ (eV)	Effective mass			*ε*_s_	Exciton property	
		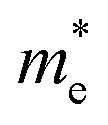	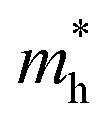	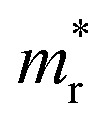		*E*_b_ (meV)	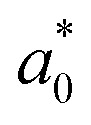 (nm)
Cs_2_AgInF_6_	4.74	0.409	1.332	0.313	2.170	904	0.367
Cs_2_AgInCl_6_	3.02 (3.3^a^)	0.272 (0.29^a^)	0.520 (0.28^a^)	0.178	2.760	319	0.819
Cs_2_AgInBr_6_	1.78 (1.5^b^)	0.161	0.381	0.113	3.750	110	1.750
Cs_2_AgInI_6_	0.06	0.106	0.297	0.078	7.321	20	4.958
Cs_2_AgSbF_6_	4.94	0.588	0.760	0.332	2.119	1005	0.338
Cs_2_AgSbCl_6_	3.32 (3.43^c^)	0.354	0.429	0.194	2.639 (4.77^c^)	379 (434^c^)	0.719 (0.56^c^)
Cs_2_AgSbBr_6_	2.30 (2.74^c^)	0.269	0.373	0.156	3.345 (5.96^c^)	190 (247^c^)	1.132 (0.76^c^)
Cs_2_AgSbI_6_	1.40	0.194	0.325	0.121	4.634	77	2.020

a Experiment and PBE0 calculation.^51^

b Experiment.^68^

c GW calculation.^30^

**Fig. 5:**
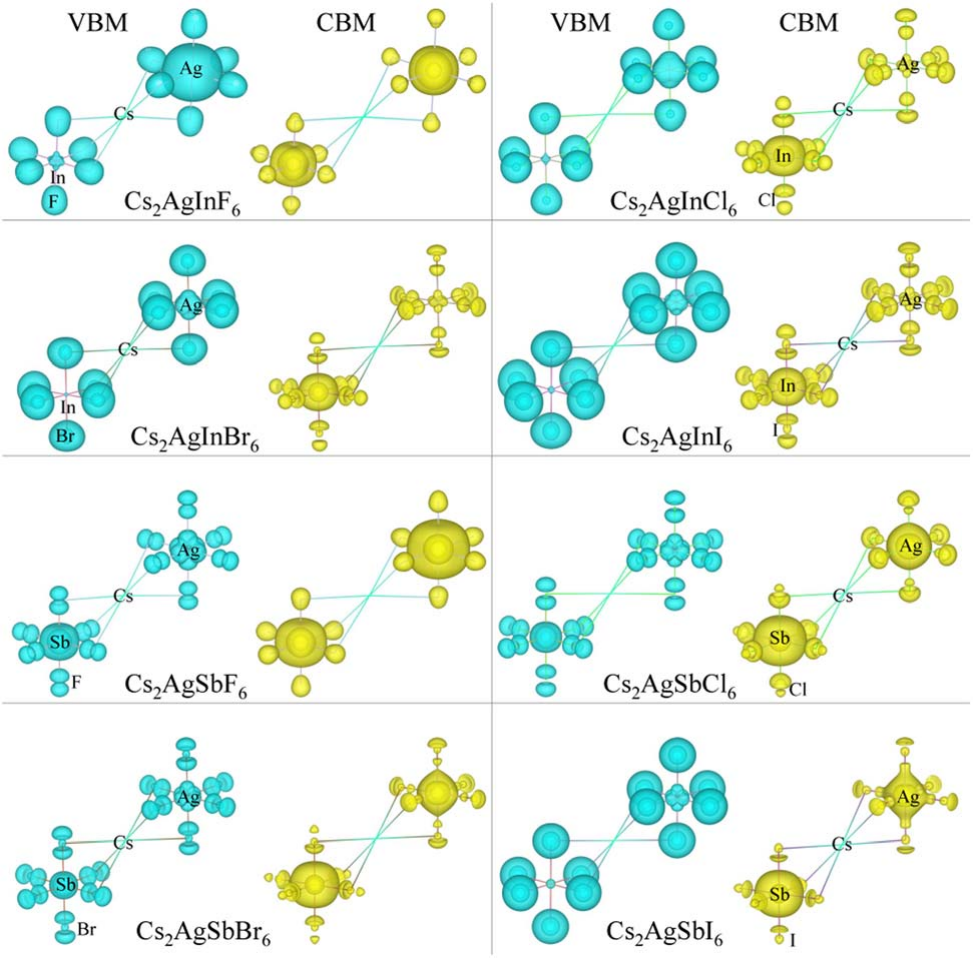
Isosurface view of integrated local density of states from the valence band maximum (VBM) energy to (VBM −1 eV) energy and from the conduction band minimum (CBM) energy to (CBM +1 eV) energy for halide double perovskites Cs_2_AgBX_6_ (B = In, Sb; X = F, Cl, Br, I).

As varying the halide component from F to I, the band gaps were found to decrease from 4.74 to 0.06 eV for the In-based HDPs and from 4.94 to 1.40 eV for the Sb-based HDPs, respectively. To clarify the reason for such variation tendency of band gaps, the electronic DOSs were calculated and analyzed (see Fig. S3, ESI†). In Fig. 5, we show the integrated local density of states (ILDOS) obtained by integrating the square of wave functions with eigen energies from the (VBM − 1 eV) energy to the VBM energy and from the CBM energy to the (CBM + 1 eV) energy. For the In-based HDPs, the top of valence band was mainly composed of Ag-4d and X-p states with a small amount of hybrid In-4d and -5p states, while the bottom of the conduction band was derived from In-5s and Ag-5s states with a certain amount of hybrid X-s and -p states (see Fig. S4, ESI†). For the cases of Sb-based HDPs, the Ag-4d, Sb-5s and X-p states comprise the VBM state, while the CBM state is composed of Ag-5s, Sb-5p and hybrid X-s and -p states. From the calculated band gaps, it is said that the fluorides and chlorides are not proper for solar cell applications due to their much higher band gaps over 3 eV, while the bromides (and possibly iodide for Sb-based HDP) can be promising candidates as solar light absorbers thanks to their suitable band gaps around 1.5 eV.

To get an insight into charge carrier transport, we calculated the effective masses of electron and hole by post-processing the band structures as follows,7
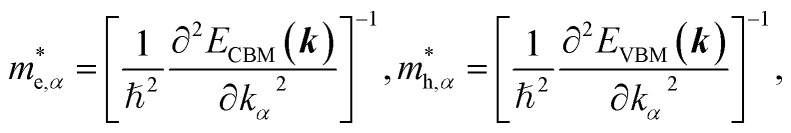
where *α*(= *x*, *y*, *z*) is the Cartesian component, and *E*_CBM_(***k***) and *E*_VBM_(***k***) are the eigen energies as functions of wave number vector ***k*** at CBM and VBM, respectively. For the cases of In-based HDPs, the effective mass vectors were found to be isotropic for electron 
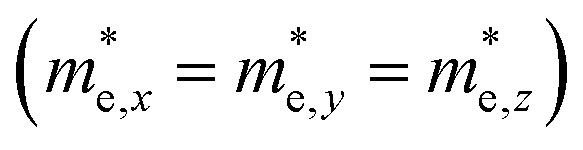
 but partially anisotropic for hole 
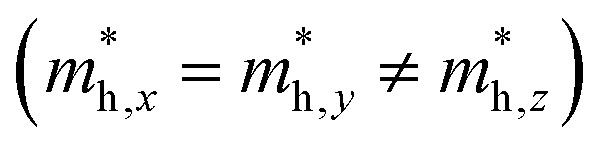
, estimated at the *Γ* point of BZ. Meanwhile, those were fully anisotropic for electron and hole 
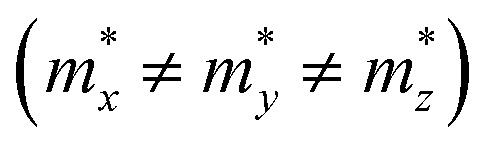
 for the cases of Sb-based ones, calculated at the *L* and *X* points, respectively. The highly anisotropy of the hole effective mass in the Sb-based perovskites is mainly related with its unusual heavy hole along the *X*(1/2 0 1/2)–*L*(1/2 1/2 1/2) line in the Brillouin zone. The anisotropy of effective masses indicates that the charge carrier mobility is different according to the direction, resulting in the negative effect on the optoelectronic properties. It is worth noting that the inclusion of the effective mass anisotropy increases the Wannier–Mott (WM) exciton binding energy by ∼20% for the Sb-based HDPs.^30^Table 3 lists the harmonic mean values of the masses along the three principal components. The reduced effective masses were also evaluated by using 
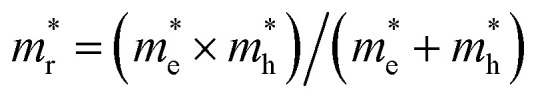
. One can see that for both In- and Sb-based HDPs the effective masses are gradually reduced as going from F to I and those of electrons are smaller than those of holes. The direct transitional In-based HDPs have smaller values of effective masses overall than the indirect transitional Sb-based compounds, indicating that the former cases are more beneficial to the charge carrier transport than the latter cases.

### 3.4 Optical properties

Then, we calculated the macroscopic dielectric functions, *ε*(*ω*) = *ε*_1_(*ω*) + *iε*_2_(*ω*), as a function of light frequency *ω*. Here, *ε*_1_(*ω*) and *ε*_2_(*ω*) are the real and imaginary parts of MDF, respectively. For high reliability, we applied the approach of solving BSE with considering the excitonic effects (denoted as BSE-EXC) and also provided the MDFs obtained with the Kohn–Sham and GW energies within RPA (denoted as KS-RPA and GW-RPA, respectively) for comparison. Once the MDFs were obtained, we could estimate the photoabsorption coefficients *α*(*ω*) and reflectivity *R*(*ω*) using the following equations,8
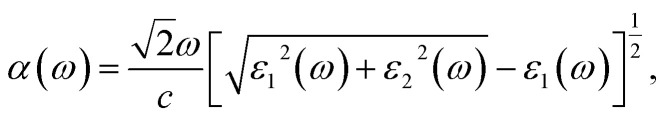
9
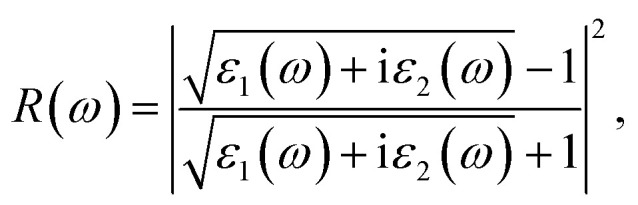
where *c* is the light velocity in vacuum.

**Fig. 6:**
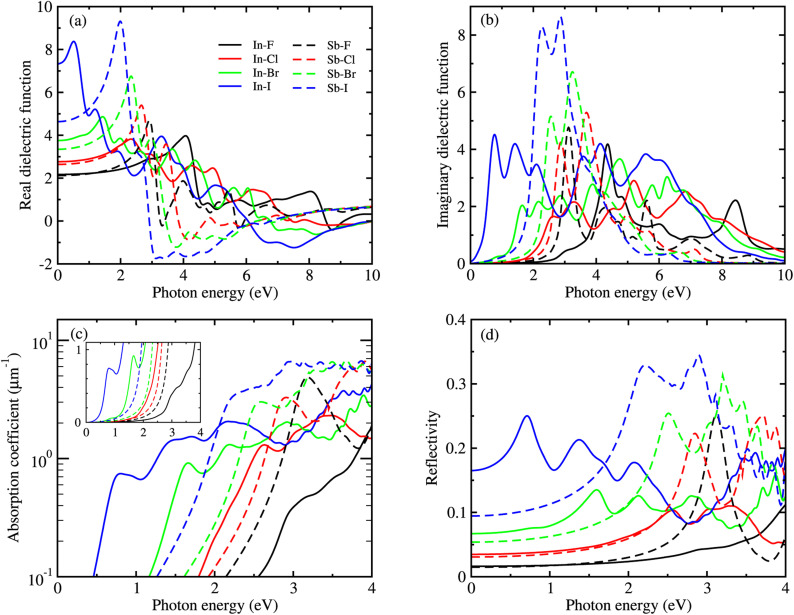
(a) Real and (b) imaginary parts of macroscopic dielectric functions, (c) photo-absorption coefficients, where the inset shows the curves with the linear scale in *y*-axis, and (d) reflectivity as functions of photon energy, calculated by solving the Bethe–Salpeter equation with considering the excitonic effect for HDPs Cs_2_AgBX_6_ (B = In, Sb; X = F, Cl, Br, I).

Fig. 6 shows the real and imaginary parts of MDFs, and the photo-absorption coefficients and reflectivity curves for the In- and Sb-based HDPs, calculated with BSE-EXC approach (see Fig. S5 and S6 for KS-RPA and GW-RPA, ESI†). In Fig. 6(a) we see that as varying the halide composition like F → Cl → Br → I, the position of the highest first peak was gradually shifted like 4.1 → 2.3 → 1.4 → 0.5 eV for the In-based compounds and 2.9 → 2.7 → 2.3 → 2.0 eV for the Sb-based HDPs. The static dielectric constants, *ε*_s_ = *ε*_1_(0) (see Table 3), and the highest first peak values were also found to gradually increase as going from F to I. These indicate that as going from F to I the Coulomb interaction between electrons and holes becomes weaker and thus the charge separation can be accelerated by reduction of exciton binding energy for both In- and Sb-based HDPs. The In-based compounds with direct band gaps showed wider position range and lower values of the highest first peaks than the Sb-based counterparts with indirect band gaps. From the photo-absorption spectra shown in Fig. 6(c), it was revealed that the absorption onset and the first peak (indicating the excitonic effect, *i.e.*, electron–hole interaction^30^) gradually shifted to a higher photon energy, *i.e.*, a shorter wavelength light, as going from X = I to F for both In- and Sb-based compounds. Such a shift of the absorption onsets is in accordance with the rise of band gap in these HDPs with a decrease of atomic number of halogen element. It should be emphasized that the Br- and Cl-based perovskites have favourable onsets and absorption coefficient for solar cell applications. In Fig. 6(d), the reflectivity was found to descend gradually as going from X = I to F, conversely indicating a slight enhancement of light absorption.

In addition, we investigated the exciton properties by calculating the exciton binding energy (*E*_b_) and exciton radius 
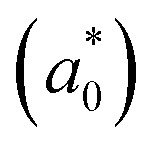
 using the hydrogenic WM model as follows,10
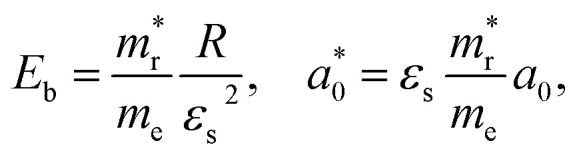
where *R* = 13.6057 eV is the Rydberg energy constant and *a*_0_ = 0.5292 Å is the Bohr radius constant. Table 3 lists the calculated values of exciton properties. For the cases of Cs_2_AgSbCl_6_ and Cs_2_AgSbBr_6_, our calculations gave the underestimated values of *E*_b_ (379 and 190 meV) and the overestimated values of 
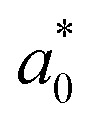
 (0.72 and 1.13 nm) when compared with the previous GW calculation^30^ (434 and 247 meV; 0.56 and 0.76 nm).

As pointed out by Biega *et al.*,^30^ such discrepancy is mainly due to that the band structures of the indirect band gap Sb-based HDPs deviate considerably from the parabolic feature and thus the anisotropic effective masses. For the cases of the indirect band gap Sb-based HDPs, the excitons were found to be highly localized within the crystal lattice,^30^ being deviated from the WM model for the weak excitons. The probability distribution of the excitonic wave functions can be estimated from the KS wave functions for the electrons and holes and the expansion coefficients for the excitonic states that can be calculated directly from the BSE outputs,^30^ and the exciton's radius can be calculated from the electron–hole correlation function,^80^ giving larger exciton binding energy and smaller exciton radius. However, we note that, when compared with the previous work for Sb–Cl and Sb–Br compounds,^30^ the discrepancies in exciton binding energy (∼55 meV) were somewhat very smaller than those (250 and 130 meV) in the previous work. It was found that Cs_2_AgSbBr_6_ exhibited a more delocalized exciton than Cs_2_AgBiBr_6_ (the same indirect band gap HDPs) due to the reduced Sb-p contribution to the CBM compared with Bi-p contribution, considering that the average electron–hole separation scales with the fractional contribution of the B-p character to the CBM.^30^ Since the main point in the present work is the variation tendency of optoelectronic properties as varying the B (In or Sb) and halogen element, we proceed the discussion with the WM model calculation to make consistency in analysis.

The lower exciton binding energy and larger exciton radius indicate the less interaction between the photo-generated charge carriers of electron and hole and thus faster dissociation, which is favourable for the solar cell applications. We found that as going from F to I the exciton binding energies were gradually decreased from 904 to 20 meV and from 1005 to 77 meV while the exciton radii were increased from 0.37 to 4.96 nm and from 0.34 to 2.02 nm for the In- and Sb-based HDPs, respectively. This indicates that the degree of exciton localization becomes weakened as going from F to I, being consistent with the variation tendency of the effective mass.

## 4 Conclusions

In this work we have investigated the structural, elastic, dynamical, electronic and optical properties of all-inorganic halide double perovskites Cs_2_AgBX_6_ (B = In, Sb; X = F, Cl, Br, I) using the first-principles calculations. Aiming at finding possibility of using them as photoabsorber for perovskite solar cell applications, we provided the variation tendencies of material properties as varying the halogen atom from F to I and the systematic comparison between the In- and Sb-based compounds. We confirmed that the cubic perovskite phase can be formed safely for all these compounds due to their proper Goldschmidt tolerance factors varying between 0.909 and 0.993 and the octahedral factors varying between 0.434 and 0.733, being placed in the stable ranges. Through the structural optimizations, it was found that the cubic lattice constant was gradually increased as going from F to I for both types of compounds, indicating the weakening of Ag–X and B–X interactions, and the In-based compounds had smaller lattice constants than the Sb-based counterparts, implying the stronger In–X attraction than Sb–X. In accordance with this, the elastic constants were found to systematically decrease as increasing the atomic number of halogen atom, while satisfying the mechanical stability criteria. In addition, these double perovskites were identified to be ductile due to the calculated values of Poisson's and Pugh's ratios over 0.64 and 2.08, respectively, and to be elastically anisotropic due to the anisotropy factors deviated from unity. Our calculations of phonon properties revealed that only the Cl-based compounds were dynamically stable since no soft modes were found, Br-based compounds exhibited weak soft modes, but F- and I-based had relatively large amount of soft modes, implying that they might be stable only at high temperature. With HSE hybrid functional, we calculated the electronic band structures, finding that the In-based HDPs had direct while the Sb-based HDPs have indirect transitions of electrons. As going from F to I, the band gaps were found to be decreased from 4.74 to 0.06 eV and from 4.94 to 1.40 eV for the In- and Sb-based perovskites, respectively, revealing that Cs_2_AgInBr_6_ and Cs_2_AgSbI_6_ have proper band gaps (1.78 and 1.40 eV) for solar cell applications. We then calculated the frequency-dependent macroscopic dielectric functions, finding that the static dielectric constants and the highest first peaks in the real parts of MDFs were gradually increased as going from F to I. Finally, we evaluated the exciton binding energy and exciton radius, revealing that the exciton binding strength became weaker as going from F to I.

## Author contributions

Chol-Jun Yu developed the original project and supervised the work. Chol-Jun Yu and Il-Chol Ri performed the DFT calculations and drafted the first manuscript. Hak-Myong Ri, Jong-Hyok Jang, Yun-Sim Kim and Un-Gi Jong assisted with the post-processing of calculation results and the useful discussions. All authors reviewed the manuscript.

## Conflicts of interest

There are no conflicts to declare.

## Supplementary Material

RA-013-D3RA02566G-s001
