# Supplementary material for: First-principles study on structural, electronic and optical properties of halide double perovskite Cs2AgBX6 (B = In, Sb; X = F, Cl, Br, I)
Source: RSC Adv. 2023 May 30;13(23):16012–22. doi: 10.1039/d3ra02566g (PMC10227528; doi:10.1039/d3ra02566g)
Supplement: RA-013-D3RA02566G-s001 [file RA-013-D3RA02566G-s001.pdf]

Electronic Supplementary Material (ESI) for RSC Advances.
This journal is © The Royal Society of Chemistry 2023

Electronic supplementary information – First-principles study on structural, electronic and optical properties of halide double perovskite Cs_2AgBX_6 (B = In, Sb; X = F, Cl, Br, I)

Chol-Jun Yu*, Il-Chol Ri, Hak-Myong Ri, Jong-Hyok Jang, Yun-Sim Kim and Un-Gi Jong

Computational Materials Design, Faculty of Materials Science, Kim Il Sung University, Pyongyang, PO Box 76, Democratic People's Republic of Korea

Table S1. Total energy (E_{tot}) and total energy per formula unit (fu) of elementary and binary solids with crystalline phase and space group.

Solid	Phase	Space group	Ref.	E_{tot} (Ry)	E_{tot} per fu (Ry)
Cs	fcc	$Fm\bar{3}m$	[1]	-252.261140	-63.065285
	bcc	$Im\bar{3}m$	[1]	-126.130728	-63.065364
Ag	fcc	$Fm\bar{3}m$	[2]	-1182.487992	-295.621998
In	tetragonal	$I4/MMM$	[3]	-266.963872	-133.481936
	orthorhombic	$Fmmm$	[4]	-533.916447	-133.479112
Sb	fcc	$Fm\bar{3}m$	[5]	-739.705388	-184.926347
	tetragonal	$I4/MMM$	[5]	-369.909949	-184.954974
	hexagonal	$R3mh$	[6]	-1109.711095	-184.951849
AgF	cubic	$Fm\bar{3}m$		-1376.882710	-344.220678
AgCl	cubic	$Fm\bar{3}m$	[7]	-1315.916179	-328.979045
AgBr	cubic	$Fm\bar{3}m$	[8]	-1370.075394	-342.518849
AgI	cubic	$Fm\bar{3}m$	[9]	-1445.211938	-361.302985
	hexagonal	$P63mc$	[10]	-722.632490	-361.316245
	zinc blende	$F\bar{4}3m$	[9]	-1445.267447	-361.316862
CsF	cubic	$Fm\bar{3}m$	[12]	-447.652490	-111.913122
	cubic	$Pm\bar{3}m$	[11]	-111.897148	-111.897148
CsCl	cubic	$Fm\bar{3}m$	[12]	-386.585878	-96.646470
	cubic	$Pm\bar{3}m$	[11]	-96.641591	-96.641591
CsBr	cubic	$Fm\bar{3}m$	[12]	-440.706264	-110.176566
	cubic	$Pm\bar{3}m$	[11]	-110.172869	-110.172869
CsI	cubic	$Fm\bar{3}m$	[12]	-515.767250	-128.941813
	cubic	$Pm\bar{3}m$	[11]	-128.940813	-128.940813
InF_3	monoclinic	$C12/m1$	[13]	-1118.322334	
$InCl_3$	monoclinic	$C12/m1$		-934.731573	
$InBr_3$	monoclinic	$C12/m1$		-1097.071959	
InI_3	monoclinic	$P121/c1$	[14]	-1322.317962	
SbF_3	orthorhombic	$Pnma$	[15]	-1324.036195	
$SbCl_3$	orthorhombic	$Pnma$		-1140.329477	
$SbBr_3$	orthorhombic	$Pbnm$		-1302.674655	
SbI_3	monoclinic	$P121/c1$		-1527.973965	

*Corresponding author: Chol-Jun Yu, Email: cj.yu@ryongnamsan.edu.kp

Table S2. Total energy (E_{tot}) of halide double perovskites $CsAgBX_6$ (B = In, Sb; X = F, Cl, Br, I) and elementary substances of Cs, Ag, In, Sb and X_2, and the elementary formation energy (E_f) per formula unit (fu).

Compound	E_{tot} (Ry)	E_{tot} (Ry) Cs	Ag	In/Sb	X_2	ΔE (Ry)	E_f (eV/fu)
Cs_2AgInF_6	-847.752926	-126.130728	-1182.487992	-266.963872	-96.918306	-1.7633	-23.9915
$Cs_2AgInCl_6$	-756.016824	-126.130728	-1182.487992	-266.963872	-66.573755	-1.0609	-14.4342
$Cs_2AgInBr_6$	-837.175734	-126.130728	-1182.487992	-266.963872	-93.671579	-0.9263	-12.6034
Cs_2AgInI_6	-949.782210	-126.130728	-1182.487992	-266.963872	-131.271802	-0.7321	-9.9613
Cs_2AgSbF_6	-899.126947	-126.130728	-1182.487992	-369.909949	-96.918306	-1.6643	-22.6443
$Cs_2AgSbCl_6$	-807.416302	-126.130728	-1182.487992	-369.909949	-66.573755	-0.9873	-13.4334
$Cs_2AgSbBr_6$	-888.595342	-126.130728	-1182.487992	-369.909949	-93.671579	-0.8729	-11.8764
Cs_2AgSbI_6	-1001.224795	-126.130728	-1182.487992	-369.909949	-131.271802	-0.7017	-9.5469

Table S3. Total energy (E_{tot}) of halide double perovskites $CsAgBX_6$ and the binary compounds of CsX, AgX and BX_3, and the binary formation energy (E_f) per formula unit (fu).

Compound	E_{tot} (Ry)	E_{tot} (Ry) CsX	AgX	BX_3	ΔE (Ry)	E_f (eV/fu)
Cs_2AgInF_6	-847.752926	-447.652490	-1376.882710	-1118.322334	-0.1254	-1.7064
$Cs_2AgInCl_6$	-756.016824	-386.585878	-1315.916179	-934.731573	-0.0619	-0.8428
$Cs_2AgInBr_6$	-837.175734	-440.706264	-1370.075394	-1097.071959	-0.0358	-0.4866
Cs_2AgInI_6	-949.782210	-515.767250	-1445.267447	-1322.317962	-0.0022	-0.0304
Cs_2AgSbF_6	-899.126947	-447.652490	-1376.882710	-1324.036195	-0.0710	-0.9657
$Cs_2AgSbCl_6$	-807.416302	-386.585878	-1315.916179	-1140.329477	-0.0619	-0.8429
$Cs_2AgSbBr_6$	-888.595342	-440.706264	-1370.075394	-1302.674655	-0.0547	-0.7442
Cs_2AgSbI_6	-1001.224795	-515.767250	-1445.267447	-1527.990350	-0.0267	-0.3636

Table S4. Effective mass of electron (m_e) and hole (m_h) in the three Cartesian directions (x, y, z), and their harmonic mean values calculated by $m^* = 3/(1/m_x + 1/m_y + 1/m_z)$.

	m_e				m_h			
Compound	x	y	z	m^*	x	y	z	m^*
Cs_2AgInF_6	0.4090	0.4090	0.4090	0.4090	1.1829	1.1829	1.7791	1.3317
$Cs_2AgInCl_6$	0.2716	0.2716	0.2716	0.2716	0.4626	0.4626	0.6030	0.5015
$Cs_2AgInBr_6$	0.1614	0.1614	0.1614	0.1614	0.3392	0.3392	0.5077	0.3814
Cs_2AgInI_6	0.1062	0.1062	0.1062	0.1062	0.2433	0.2433	0.4212	0.2832
Cs_2AgSbF_6	0.6062	0.5826	0.5769	0.5883	0.5974	1.0447	0.7601	0.7601
$Cs_2AgSbCl_6$	0.3646	0.3511	0.3479	0.3544	0.3382	0.5879	0.4294	0.4294
$Cs_2AgSbBr_6$	0.2803	0.2659	0.2625	0.2693	0.2911	0.5181	0.3728	0.3728
Cs_2AgSbI_6	0.2088	0.1895	0.1852	0.1940	0.2536	0.4508	0.3246	0.3246

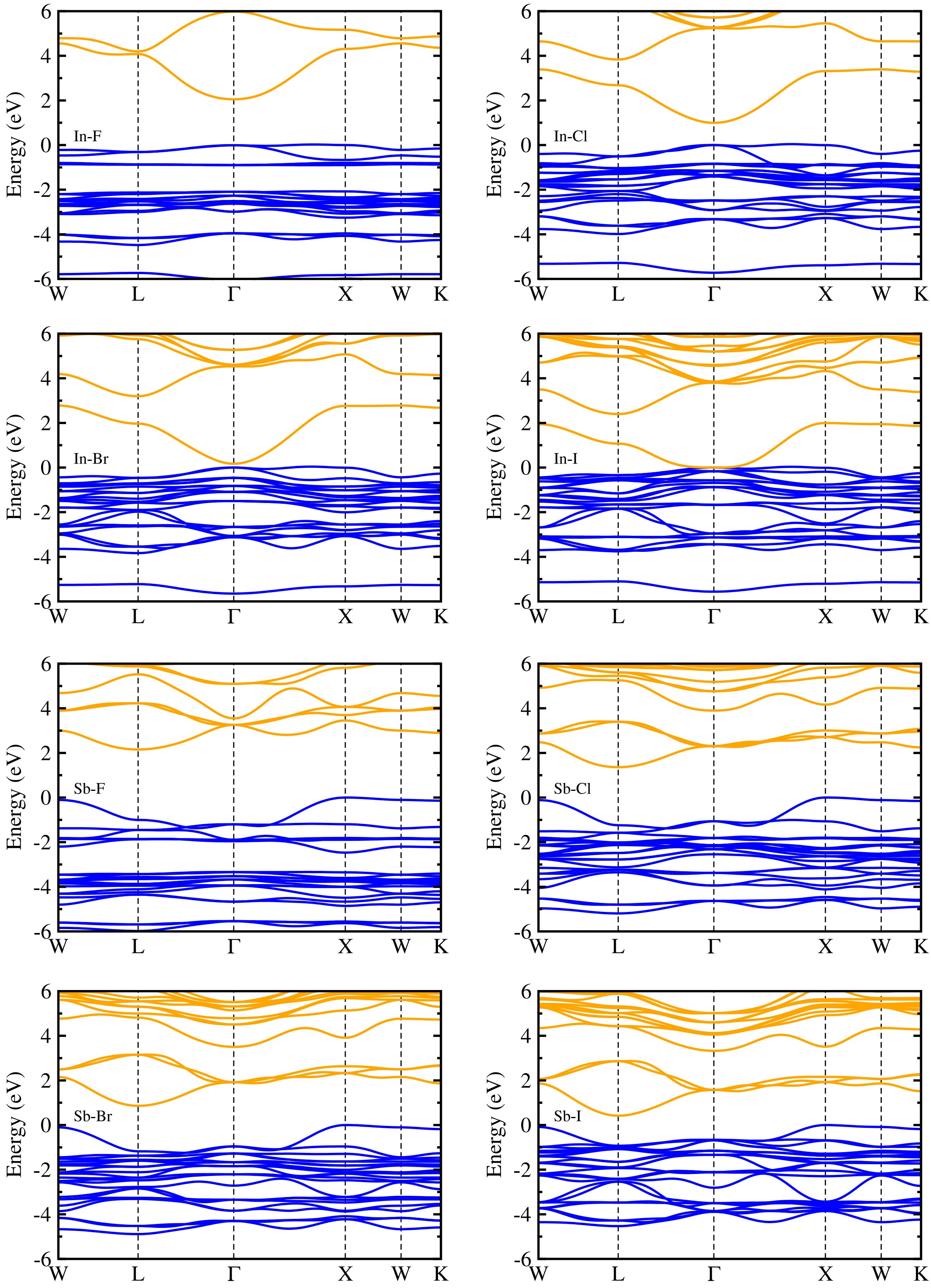

Figure S1. Electronic band structures of all-inorganic halide double perovskites Cs_2AgBX_6 (B = In, Sb; X = F, Cl, Br, I), calculated with PBE functional.

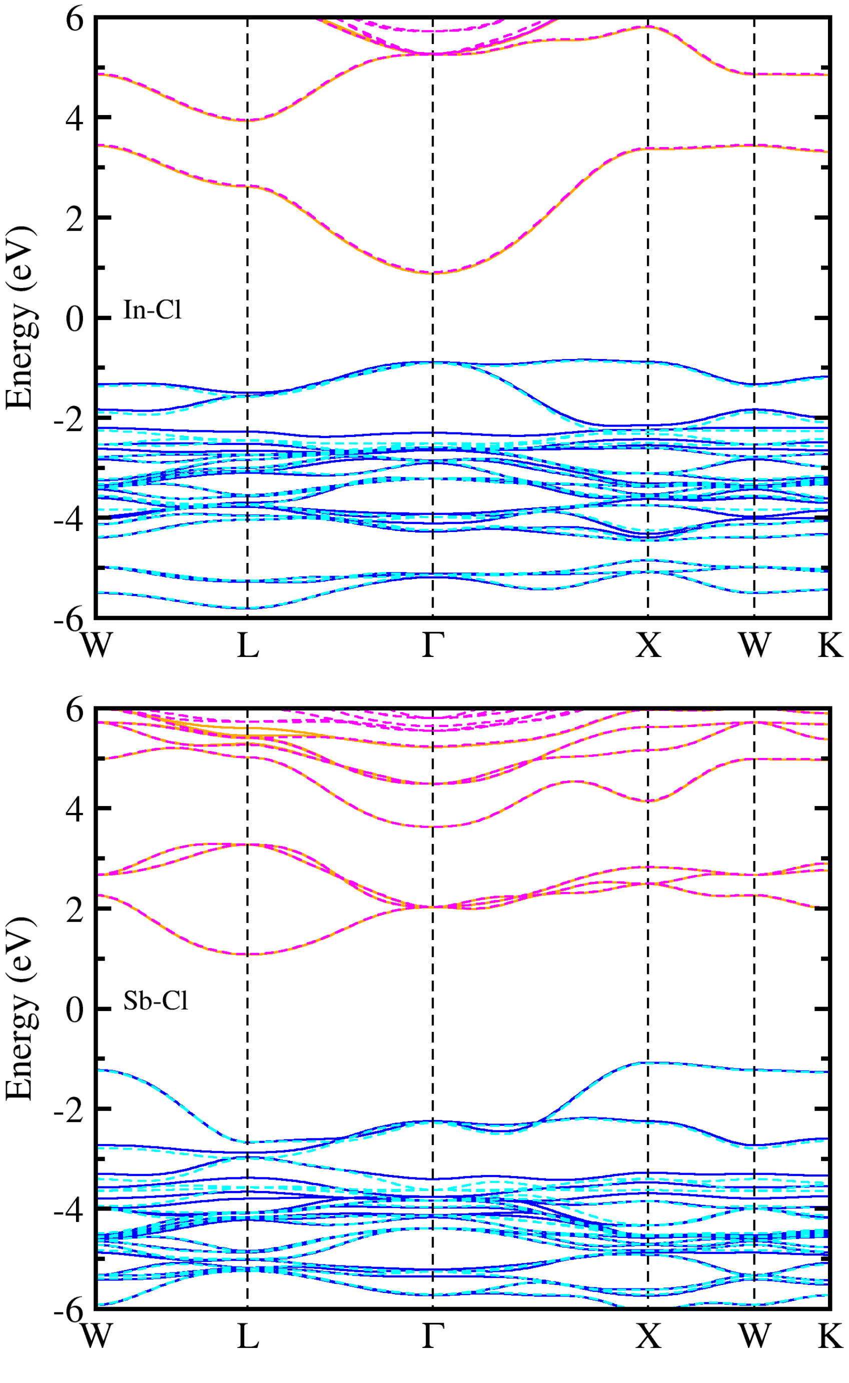

Figure S2. Electronic band structures of all-inorganic halide double perovskites $Cs_2AgInCl_6$ (top) and $Cs_2AgSbCl_6$ (bottom), calculated with PBE (solid lines) and PBE+SOC (dashed lines).

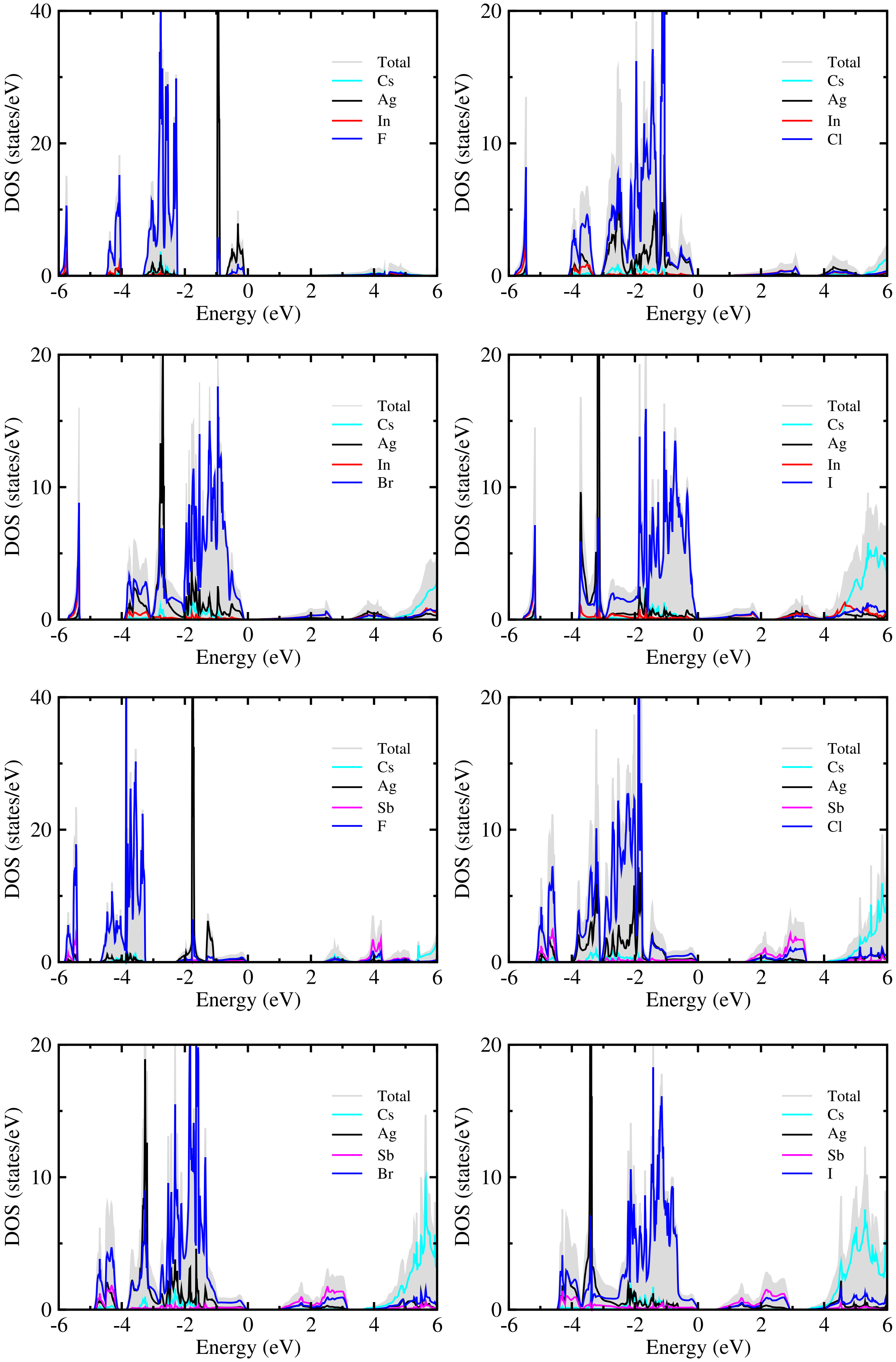

Figure S3. Atom-projected total density of states (DOS) of all-inorganic halide double perovskites Cs_2AgBX_6 (B = In, Sb; X = F, Cl, Br, I), calculated with HSE hybrid functional.

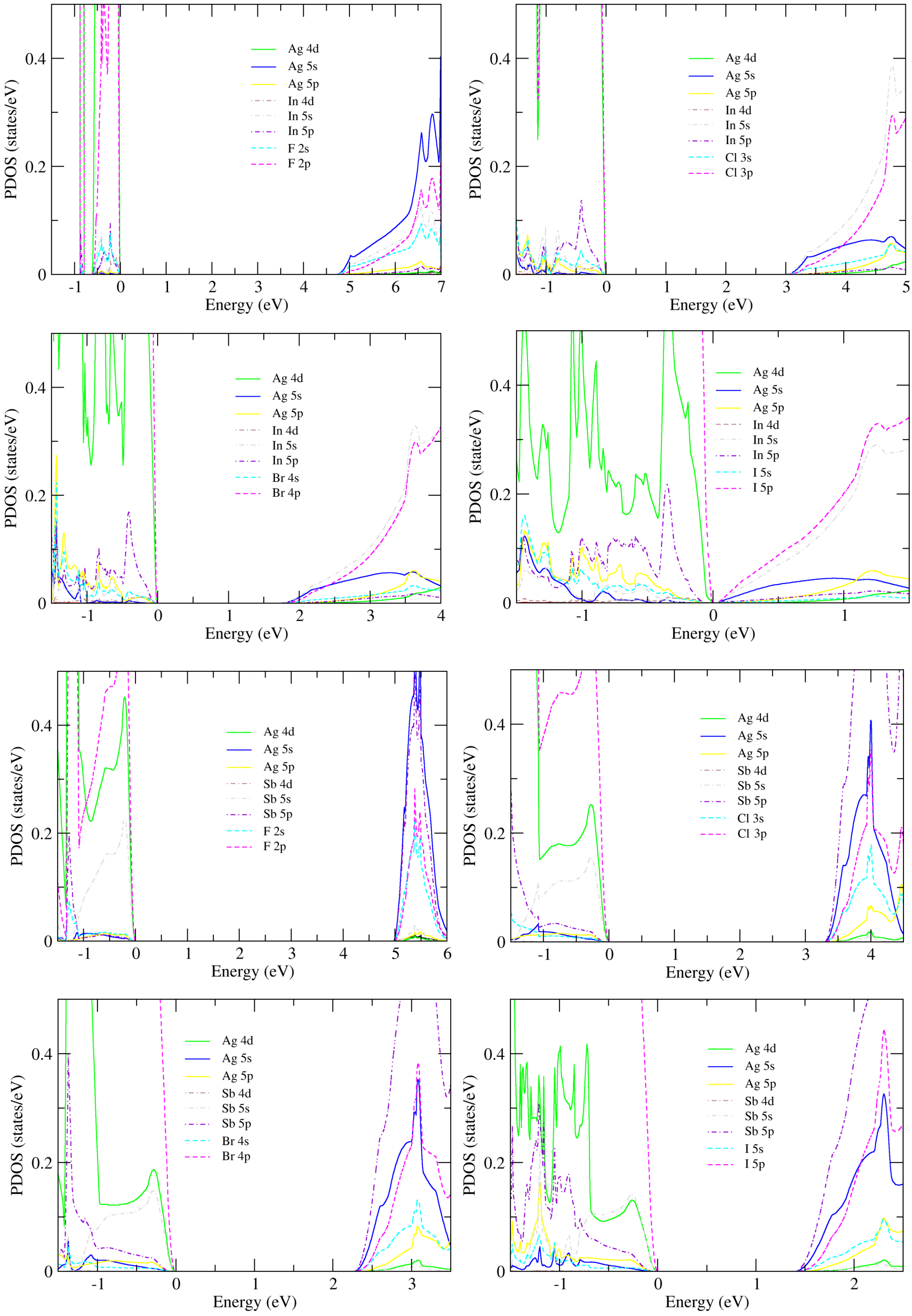

Figure S4. Orbital-resolved partial density of states (PDOS) of all-inorganic halide double perovskites Cs_2AgBX_6 (B = In, Sb; X = F, Cl, Br, I), calculated with HSE hybrid functional.

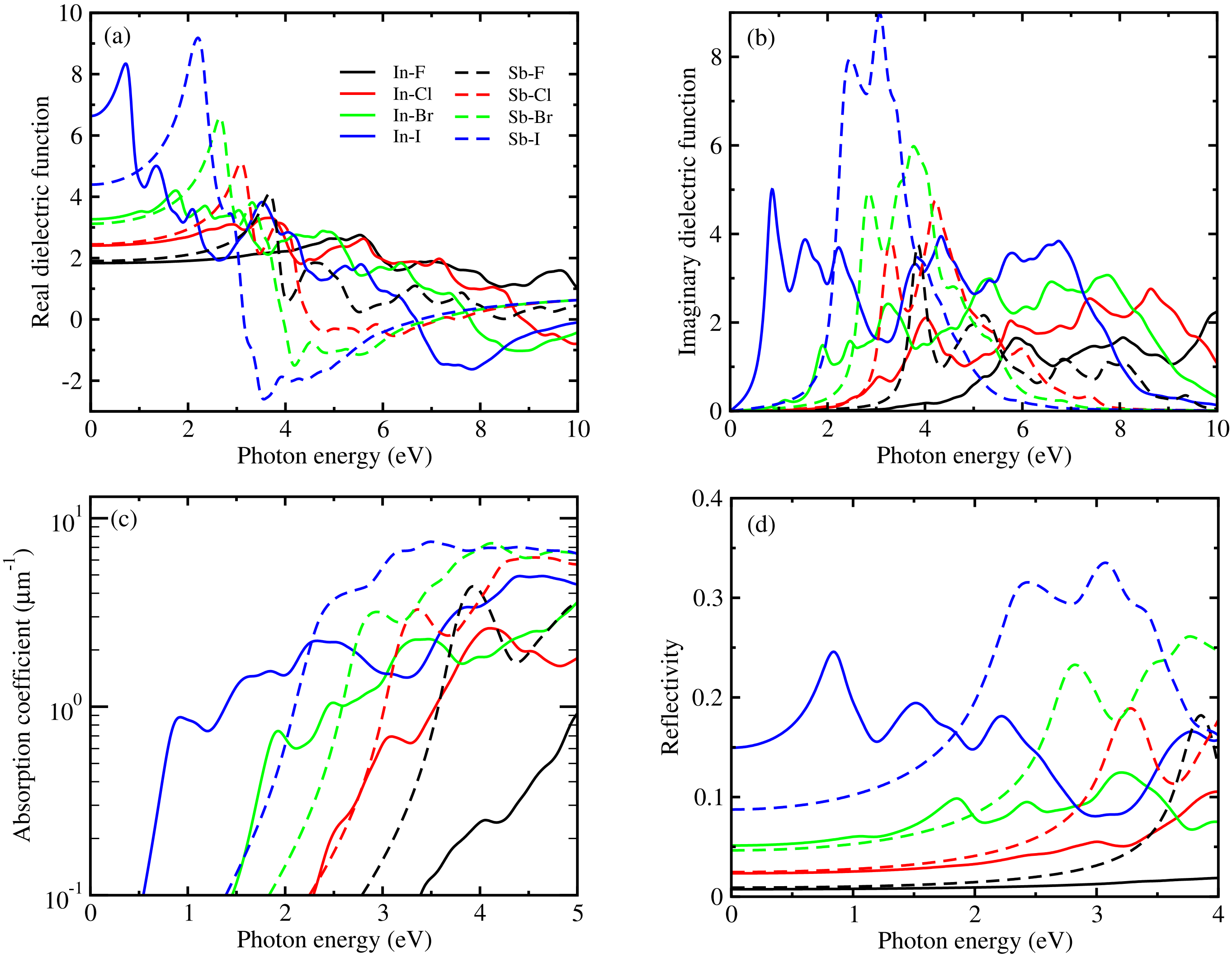

Figure S5. (a) Real and (b) imaginary parts of macroscopic dielectric functions, (c) photo-absorption coefficients, and (d) reflectivity as functions of photon energy for Cs_2AgBX_6 (B = In, Sb; X = F, Cl, Br, I), calculated with the *GW* energies within RPA (i.e., *GW*-RPA).

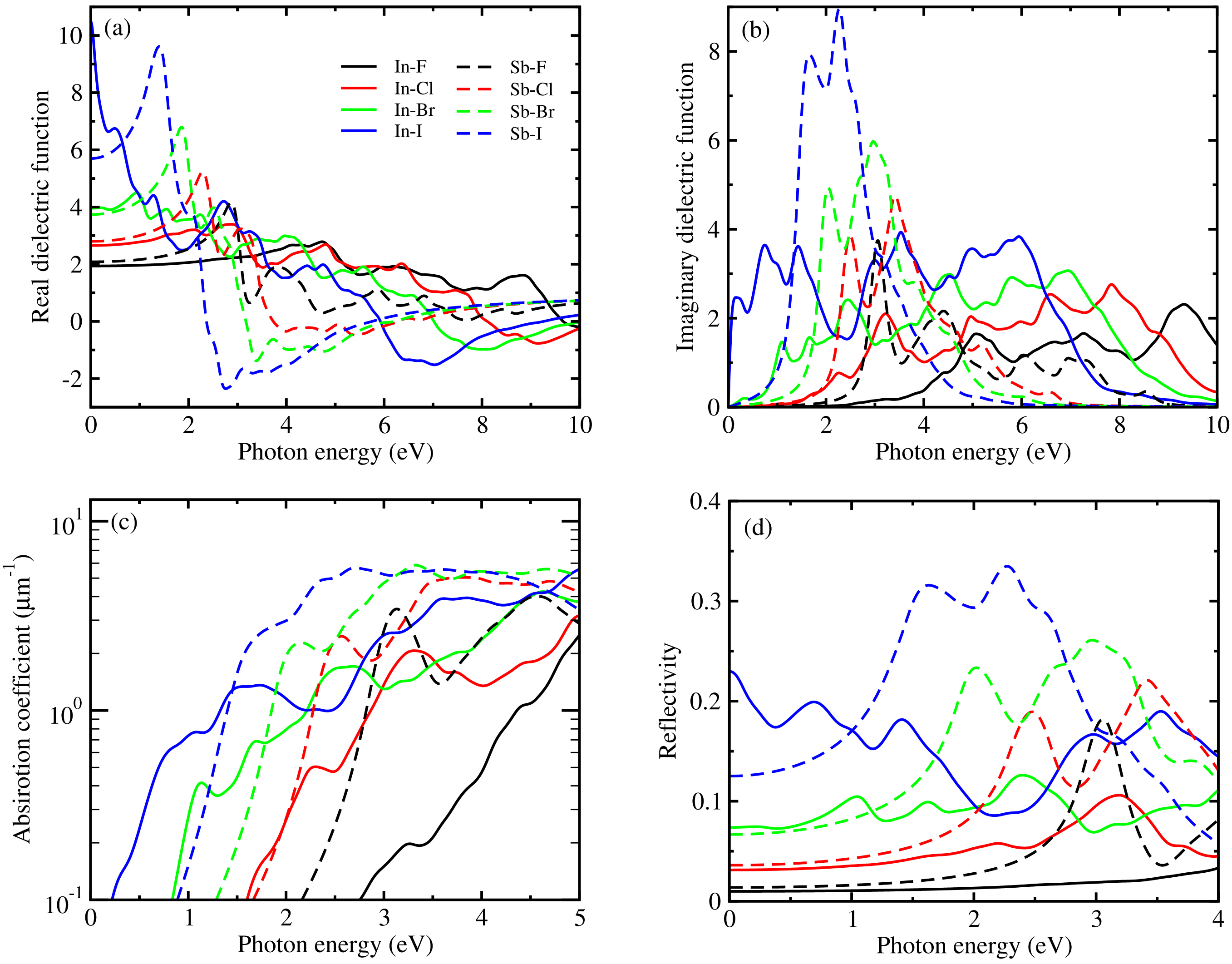

Figure S6. (a) Real and (b) imaginary parts of macroscopic dielectric functions, (c) photo-absorption coefficients, and (d) reflectivity as functions of photon energy for Cs_2AgBX_6 (B = In, Sb; X = F, Cl, Br, I), calculated with the Kohn-Sham energies within RPA (i.e., KS-RPA).

References

[1] C. E. Weir, G. J. Piermarini, S. Block, On the crystal structures of Cs II and Ga II, *J. Chem. Phys.* **1971**, *54*, 2768–2770.

[2] I.-K. Suh, H. Ohta, Y. Waseda, High-temperature thermal expansion of six metallic elements measured by dilatation method and X-ray diffraction, *J. Mater. Sci.* **1988**, *23*, 757–760.

[3] E. G. Moshopoulou, R. M. Ibberson, J. L. Sarrao, J. D. Thompson, Z. Fisk, Structure of Ce_2RhIn_8: an example of complementary use of high-resolution neutron powder diffraction and reciprocal-space mapping to study complex materials, *Acta Crystal. B* **2006**, *62*, 173–189.

[4] K. Takemura, H. Fujihaza, High-pressure structural phase transition in indium, *Phys. Rev. B* **1993**, *47*, 8465–8470.

[5] D. Akhtar, V. D. Vankar, T. C. Goel, K. L. Chopra, Metastable structures of liquid-quenched and vapour-quenched antimony films, *J. Mater. Sci.* **1979**, *14*, 988–994.

[6] J. Q. Li, X. W. Feng, M. A. Sun, W. Q. Ao, F. S. Liu, Y. Du, Solvothermal synthesis of nano-sized skutterudite $Co_{4-x}Fe_xSb_{12}$ powders, *Mater. Chem. Phys.* **2008**, *112*, 57–62.

[7] S. Hull, D. A. Keen, Pressure-induced phase transitions in AgCl, AgBr, and AgI, *Phys. Rev. B* **1999**, *59*, 750–761.

[8] T. Benmessabih, B. Amrani, F. El haj Hassan, F. Hamdache, M. Zoaeter, Computational study of AgCl and AgBr semiconductors, *Physica B* **2007**, *392*, 309–317.

[9] B. Amrani, R. Ahmed, F. El haj Hassan, A. H. Reshak, Structural, electronic and optical properties of AgI under pressure, *Phys. Lett. A* **2008**, *372*, 2502–2508.

[10] A. Yoshiasa, K. Koto, K. Kanamaru, S. Emura, H. Horiuchi, Anharmonic thermal vibrations in wurtzite-type AgI, *Acta Crystal. B* **1987**, *43*, 434–440.

[11] P. Cortona, Direct determination of self-consistent total energies and charge densities of solids: A study of the cohesive properties of the alkali halides, *Phys. Rev. B* **1992**, *46*, 2008–2014.

[12] M. Blackman, I. H. Khan, The polymorphism of thallium and other halides at low temperatures, *Proc. Phys. Soc. London* **1961**, *77*, 471–475.

[13] C. Hebecker, R. Hoppe, Zur Kristallstruktur von Indium- und Thallium trifluorid, *Naturwissenschaften* **1966**, *53*, 104.

[14] J. D. Forrester, A. Zalkin, D. H. Templeton, Crystal and Molecular Structure of Indium(III) Iodide (In_2I_6), *Inorg. Chem.* **1964**, *3*, 63–67.

[15] A. J. Edwards, Fluoride crystal structures. Part XIV. Antimony trifluoride: A redetermination, *J. Chem. Soc. A* **1970**, *1970*, 2751–2753.
